# (*E*)-4-(4-Hydr­oxy-3-methoxy­benzyl­idene­amino)-3-[1-(4-isobutyl­phen­yl)eth­yl]-1*H*-1,2,4-triazole-5(4*H*)-thione

**DOI:** 10.1107/S160053680905209X

**Published:** 2009-12-09

**Authors:** Jia Hao Goh, Hoong-Kun Fun, A. C. Vinayaka, B. Kalluraya

**Affiliations:** a X-ray Crystallography Unit, School of Physics, Universiti Sains Malaysia, 11800 USM, Penang, Malaysia; b Department of Studies in Chemistry, Mangalore University, Mangalagangotri, Mangalore 574 199, India

## Abstract

The asymmetric unit of the title compound, C_22_H_26_N_4_O_2_S, contains two crystallographically independent mol­ecules (*A* and *B*). The isobutyl unit of mol­ecule *B* is disordered over two orientations with refined occupancies of 0.785 (6) and 0.215 (6). In each mol­ecule, intra­molecular C—H⋯S hydrogen bonds generate *S*(6) ring motifs. The essentially planar 1,2,4-triazole rings [r.m.s. deviations of 0.004 (2) and 0.011 (2) Å, in *A* and *B* respectively] form dihedral angles of 85.86 (12), 8.38 (10)°, respectively, with the isobutyl-substituted phenyl ring and the 2-methoxy­phenol substituent in mol­ecule *A* [89.26 (13) and 2.46 (10)°, respectively, in *B*]. In the crystal structure, inter­molecular N—H⋯N and N—H⋯S hydrogen bonds link neighbouring mol­ecules, generating *R* ^2^_2_(7) ring motifs. These molecules are further inter­connected into extended chains along [20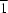] by inter­molecular O—H⋯O hydrogen bonds. The crystal structure is further stabilized by π–π [centroid-centroid distance = 3.6299 (13) Å] and C—H⋯π inter­actions. A short O⋯O contact of 2.781 (2) Å is also observed.

## Related literature

For general background to and applications of the title compound, see: Bekircan & Bektas (2006 ▶); Fun *et al.* (2009 ▶); Koparır *et al.* (2005 ▶). For hydrogen-bond motifs, see: Bernstein *et al.* (1995 ▶). For bond-length data, see: Allen *et al.* (1987 ▶). For a closely related 1,2,4-triazole structure, see: Fun *et al.* (2009 ▶). For the stability of the temperature controller used for the data collection, see: Cosier & Glazer (1986 ▶).
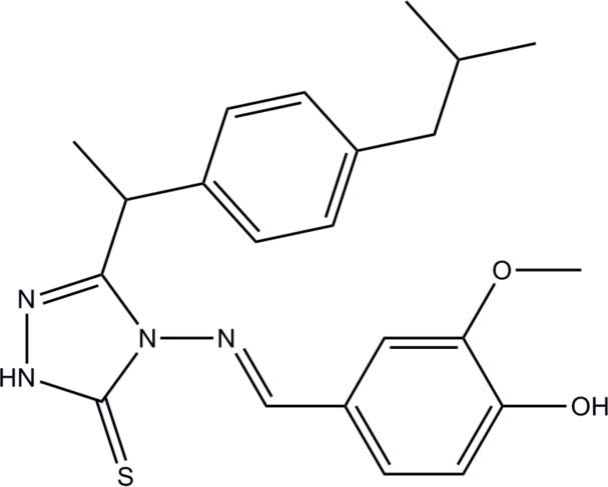


## Experimental

### 

#### Crystal data

C_22_H_26_N_4_O_2_S
*M* *_r_* = 410.53
Triclinic, 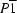

*a* = 9.8646 (3) Å
*b* = 14.2026 (5) Å
*c* = 16.6758 (6) Å
α = 69.048 (2)°
β = 79.881 (2)°
γ = 85.946 (2)°
*V* = 2147.83 (13) Å^3^
*Z* = 4
Mo *K*α radiation
μ = 0.18 mm^−1^
*T* = 100 K
0.31 × 0.22 × 0.15 mm

#### Data collection

Bruker SMART APEXII CCD area-detector diffractometer
Absorption correction: multi-scan (*SADABS*; Bruker, 2005 ▶) *T*_min_ = 0.948, *T*_max_ = 0.974
39640 measured reflections
7435 independent reflections
5503 reflections with *I* > 2σ(*I*)
*R*_int_ = 0.060

#### Refinement

*R*[*F* ^2^ > 2σ(*F* ^2^)] = 0.044
*wR*(*F* ^2^) = 0.111
*S* = 1.04
7435 reflections
571 parameters
H atoms treated by a mixture of independent and constrained refinement
Δρ_max_ = 0.25 e Å^−3^
Δρ_min_ = −0.25 e Å^−3^

### 

Data collection: *APEX2* (Bruker, 2005 ▶); cell refinement: *SAINT* (Bruker, 2005 ▶); data reduction: *SAINT*; program(s) used to solve structure: *SHELXTL* (Sheldrick, 2008 ▶); program(s) used to refine structure: *SHELXTL*; molecular graphics: *SHELXTL*; software used to prepare material for publication: *SHELXTL* and *PLATON* (Spek, 2009 ▶).

## Supplementary Material

Crystal structure: contains datablocks global, I. DOI: 10.1107/S160053680905209X/sj2704sup1.cif

Structure factors: contains datablocks I. DOI: 10.1107/S160053680905209X/sj2704Isup2.hkl

Additional supplementary materials:  crystallographic information; 3D view; checkCIF report

## Figures and Tables

**Table 1:** Hydrogen-bond geometry (Å, °)

*D*—H⋯*A*	*D*—H	H⋯*A*	*D*⋯*A*	*D*—H⋯*A*
N3*A*—H1*N*3⋯N4*B*^i^	0.82 (3)	2.19 (3)	2.968 (3)	161 (3)
N3*B*—H2*N*3⋯S1*A*^ii^	0.87 (3)	2.39 (3)	3.227 (2)	161 (3)
O2*A*—H1*O*2⋯O2*B*^iii^	0.75 (3)	2.11 (3)	2.808 (3)	157 (3)
C7*A*—H7*AA*⋯S1*A*	0.93	2.43	3.193 (2)	139
C7*B*—H7*BA*⋯S1*B*	0.93	2.53	3.243 (2)	134
C5*B*—H5*BA*⋯*Cg*1^iv^	0.93	2.91	3.660 (3)	139

Symmetry codes: (i) 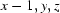; (ii) 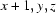; (iii) 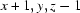; (iv) 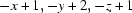. *Cg*1 is the centroid of the C11*A*—C16*A* phenyl ring.

**Fig. 1.**
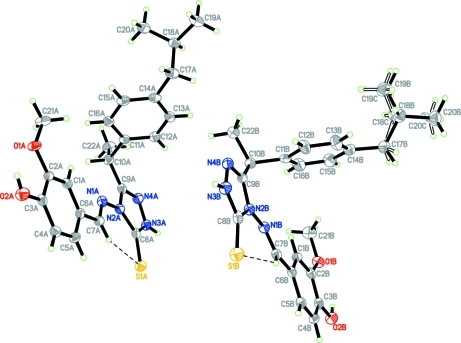
The asymmetric unit of the title compound, showing 50% probability displacement ellipsoids for non-H atoms and the atom-numbering scheme. Intramolecular hydrogen bonds are shown as dashed lines. Open bonds indicate the minor disorder component.

**Fig. 2.**
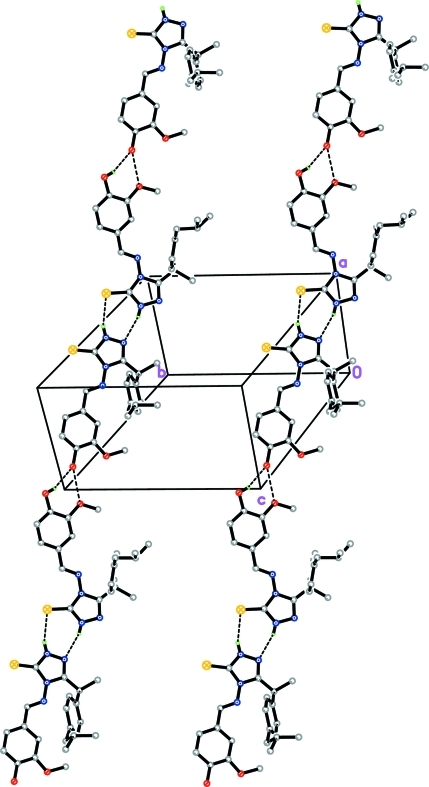
The crystal structure of the title compound, showing R22(7) ring motifs being linked into one-dimensional extended chains. Only the major disorder component is shown. H atoms not involved in intermolecular interactions (dashed lines) have been omitted for clarity.

## supplementary crystallographic information

### Comment

1,2,4-triazoles and their derivatives are found to be associated with various biological activities with for example anti-convulsant, anti-fungal, anti-cancer, anti-inflammatory, anti-bacterial properties (Bekircan & Bektas, 2006) and also act as effective pesticides (Koparır *et al.*, 2005). Several compounds containing 1,2,4-triazole rings are well known as drugs. Furthermore, in recent years, some Schiff base derivatives of 1,2,4-triazoles have been found to possess pharmacological activities (Fun *et al.*, 2009). As part of our ongoing work on Schiff base derivatives, we report here the crystal structure of this new Schiff base.

In the asymmetric unit of the title 1,2,4-triazole compound, there are two crystallographically independent molecules, designated *A* and *B* (Fig. 1). In molecule *B*, the isobutyl unit is disordered over two positions with a refined site-occupancy ratio of 0.785 (6):0.215 (6). In each molecule, intramolecular C7A—H7AA···S1A and C7B—H7BA···S1B hydrogen bonds (Table 1) generate six-membered rings, producing *S*(6) ring motifs (Fig. 1, Bernstein *et al.*, 1995). The 1,2,4-triazole rings (N2/C8/N3/N4/C9) are essentially planar, with maximum deviations of -0.004 (2) and -0.011 (2) Å, respectively, for atoms C8A and C8B. In molecule *A*, the 1,2,4-triazole ring makes dihedral angles of 85.86 (12) and 8.38 (10)°, respectively, with isobutyl-substituted phenyl ring (C11-C16) and 2-methoxyphenol moiety (C1-C6/C21/O1/O2); the comparable angles for molecule *B* are 89.26 (13) and 2.46 (10)°, respectively. The bond lengths (Allen *et al.*, 1987) and angles are within normal ranges and comparable to a closely related structure (Fun *et al.*, 2009).

In the crystal structure (Fig. 2), intermolecular N3A—H1N3···N4B and N3B—H2N3···S1A hydrogen bonds (Table 1) link neighbouring molecules into *R*^2^_2_(7) ring motifs (Bernstein *et al.*, 1995). Intermolecular O2A—H1O2···O2B hydrogen bonds (Table 1) interconnect these hydrogen bond ring motifs into one-dimensional extended chains along [201]. An interesting feature of the crystal structure is the short intermolecular O1A···O2B contacts [symmetry code: -1+x, y, 1+z] with a distance of 2.781 (2) Å, which is significantly shorter than the sum of the van der Waals radii of the oxygen atoms (3.04 Å). The crystal structure is further stabilized by intermolecular C5B—H5BA···*Cg*1 as well as *Cg*2···*Cg*3 interactions [centroid-centroid distance = 3.6299 (13) Å^iv^; *Cg*1, *Cg*2 and *Cg*3 are the centroids of C11A-C16A, C1A-C6A and C1B-C6B phenyl rings, respectively].

### Experimental

The title Schiff base compound was obtained by refluxing 4-amino-5-[1-(4-isobutylphenyl)ethyl]-4*H*-1,2,4-triazole-3-thiol (0.01 mol) and 4-hydroxy-3-methoxybenzaldehyde (0.01 mol) in ethanol (20 ml) for 6 h, with the addition of three drops of concentrated sulphuric acid. The solid product obtained was collected by filtration, washed with ethanol and dried. It was then recrystallized using ethanol. Single crystals suitable for X-ray analysis were obtained from ethanol by slow evaporation.

### Refinement

Atoms H1N3, H2N3, H1O2 and H2O2 were located from difference Fourier map and allowed to refine freely. All other hydrogen atoms were placed in their calculated positions, with C—H = 0.93 – 0.98 Å, and refined using a riding model, with *U*_iso_ = 1.2 or 1.5 *U*_eq_(C). A rotating group model was used for the methyl groups. The isobutyl unit of molecule *B* is disordered over two positions with refined occupancies of 0.785 (6) and 0.215 (6). The same U^ij^ parameters were used for the atom pair C19B/C19C. The reflection (010) was omitted as the intensity was affected by the beam backstop.

### Crystal data

**Table:**

C_22_H_26_N_4_O_2_S	*Z* = 4
*M**_r_* = 410.53	*F*(000) = 872
Triclinic, *P*1	*D*_x_ = 1.270 Mg m^−^^3^
Hall symbol: -P 1	Mo *K*α radiation, λ = 0.71073 Å
*a* = 9.8646 (3) Å	Cell parameters from 9988 reflections
*b* = 14.2026 (5) Å	θ = 2.5–29.9°
*c* = 16.6758 (6) Å	µ = 0.18 mm^−^^1^
α = 69.048 (2)°	*T* = 100 K
β = 79.881 (2)°	Block, colourless
γ = 85.946 (2)°	0.31 × 0.22 × 0.15 mm
*V* = 2147.83 (13) Å^3^	

### Data collection

**Table:**

Bruker SMART APEXII CCD area-detector diffractometer	7435 independent reflections
Radiation source: fine-focus sealed tube	5503 reflections with *I* > 2σ(*I*)
graphite	*R*_int_ = 0.060
φ and ω scans	θ_max_ = 25.0°, θ_min_ = 2.3°
Absorption correction: multi-scan (*SADABS*; Bruker, 2005)	*h* = −11→11
*T*_min_ = 0.948, *T*_max_ = 0.974	*k* = −16→16
39640 measured reflections	*l* = −19→19

### Refinement

**Table:**

Refinement on *F*^2^	Primary atom site location: structure-invariant direct methods
Least-squares matrix: full	Secondary atom site location: difference Fourier map
*R*[*F*^2^ > 2σ(*F*^2^)] = 0.044	Hydrogen site location: inferred from neighbouring sites
*wR*(*F*^2^) = 0.111	H atoms treated by a mixture of independent and constrained refinement
*S* = 1.03	*w* = 1/[σ^2^(*F*_o_^2^) + (0.0383*P*)^2^ + 1.5959*P*] where *P* = (*F*_o_^2^ + 2*F*_c_^2^)/3
7435 reflections	(Δ/σ)_max_ = 0.001
571 parameters	Δρ_max_ = 0.25 e Å^−^^3^
0 restraints	Δρ_min_ = −0.25 e Å^−^^3^

### Special details

**Table:**

Experimental. The crystal was placed in the cold stream of an Oxford Cyrosystems Cobra open-flow nitrogen cryostat (Cosier & Glazer, 1986) operating at 100.0 (1)K.
Geometry. All esds (except the esd in the dihedral angle between two l.s. planes) are estimated using the full covariance matrix. The cell esds are taken into account individually in the estimation of esds in distances, angles and torsion angles; correlations between esds in cell parameters are only used when they are defined by crystal symmetry. An approximate (isotropic) treatment of cell esds is used for estimating esds involving l.s. planes.
Refinement. Refinement of F^2^ against ALL reflections. The weighted R-factor wR and goodness of fit S are based on F^2^, conventional R-factors R are based on F, with F set to zero for negative F^2^. The threshold expression of F^2^ > 2sigma(F^2^) is used only for calculating R-factors(gt) etc. and is not relevant to the choice of reflections for refinement. R-factors based on F^2^ are statistically about twice as large as those based on F, and R- factors based on ALL data will be even larger.

### Fractional atomic coordinates and isotropic or equivalent isotropic displacement parameters (Å^2^)

**Table:**

	*x*	*y*	*z*	*U*_iso_*/*U*_eq_	Occ. (<1)
S1A	0.14118 (6)	1.03163 (5)	0.30146 (4)	0.02423 (16)	
O1A	0.82404 (16)	0.95561 (12)	−0.02253 (10)	0.0245 (4)	
O2A	0.87801 (18)	1.15369 (14)	−0.06581 (11)	0.0279 (4)	
N1A	0.36062 (18)	0.90569 (15)	0.18842 (12)	0.0211 (5)	
N2A	0.24185 (18)	0.87048 (14)	0.25021 (12)	0.0191 (4)	
N3A	0.0621 (2)	0.83823 (15)	0.34536 (13)	0.0224 (5)	
N4A	0.09167 (19)	0.75035 (15)	0.32812 (12)	0.0241 (5)	
C1A	0.6112 (2)	0.97179 (17)	0.07204 (14)	0.0197 (5)	
H1AA	0.5897	0.9042	0.0876	0.024*	
C2A	0.7294 (2)	1.01022 (17)	0.01489 (14)	0.0196 (5)	
C3A	0.7613 (2)	1.11248 (17)	−0.00986 (14)	0.0199 (5)	
C4A	0.6741 (2)	1.17468 (18)	0.02374 (15)	0.0227 (5)	
H4AA	0.6944	1.2426	0.0069	0.027*	
C5A	0.5561 (2)	1.13561 (18)	0.08250 (15)	0.0234 (6)	
H5AA	0.4986	1.1774	0.1058	0.028*	
C6A	0.5232 (2)	1.03453 (17)	0.10683 (14)	0.0196 (5)	
C7A	0.3988 (2)	0.99654 (18)	0.16938 (15)	0.0218 (5)	
H7AA	0.3472	1.0383	0.1955	0.026*	
C8A	0.1491 (2)	0.91387 (18)	0.29956 (14)	0.0202 (5)	
C9A	0.2026 (2)	0.77220 (18)	0.26948 (15)	0.0220 (5)	
C10A	0.2820 (2)	0.70098 (18)	0.23134 (16)	0.0245 (6)	
H10A	0.3051	0.7358	0.1683	0.029*	
C11A	0.4164 (2)	0.66929 (17)	0.26810 (15)	0.0213 (5)	
C12A	0.4173 (2)	0.63440 (17)	0.35689 (15)	0.0238 (6)	
H12A	0.3357	0.6333	0.3948	0.029*	
C13A	0.5383 (2)	0.60111 (17)	0.38975 (16)	0.0249 (6)	
H13A	0.5364	0.5786	0.4496	0.030*	
C14A	0.6620 (2)	0.60045 (17)	0.33603 (16)	0.0242 (6)	
C15A	0.6609 (2)	0.63709 (18)	0.24672 (16)	0.0268 (6)	
H15A	0.7429	0.6388	0.2089	0.032*	
C16A	0.5410 (2)	0.67088 (17)	0.21306 (16)	0.0245 (6)	
H16A	0.5434	0.6949	0.1531	0.029*	
C17A	0.7915 (3)	0.55792 (19)	0.37397 (17)	0.0298 (6)	
H17A	0.7869	0.5692	0.4284	0.036*	
H17B	0.8703	0.5943	0.3343	0.036*	
C18A	0.8138 (2)	0.44485 (18)	0.39067 (15)	0.0247 (6)	
H18A	0.7296	0.4097	0.4259	0.030*	
C19A	0.9313 (3)	0.40483 (19)	0.44231 (18)	0.0336 (6)	
H19A	0.9396	0.3332	0.4563	0.050*	
H19B	0.9126	0.4197	0.4950	0.050*	
H19C	1.0156	0.4364	0.4082	0.050*	
C20A	0.8408 (3)	0.42227 (19)	0.30693 (17)	0.0358 (7)	
H20A	0.8545	0.3510	0.3202	0.054*	
H20B	0.9217	0.4573	0.2707	0.054*	
H20C	0.7633	0.4442	0.2769	0.054*	
C21A	0.8043 (3)	0.84942 (18)	0.00711 (18)	0.0314 (6)	
H21A	0.8784	0.8193	−0.0214	0.047*	
H21B	0.7185	0.8360	−0.0062	0.047*	
H21C	0.8028	0.8214	0.0689	0.047*	
C22A	0.1956 (3)	0.6086 (2)	0.24750 (18)	0.0335 (6)	
H22A	0.1170	0.6294	0.2184	0.050*	
H22B	0.1654	0.5766	0.3089	0.050*	
H22C	0.2504	0.5620	0.2253	0.050*	
S1B	0.76869 (7)	1.16289 (5)	0.46186 (4)	0.02935 (17)	
O1B	0.12190 (16)	0.86906 (12)	0.82309 (10)	0.0249 (4)	
O2B	0.03960 (17)	1.04132 (15)	0.84371 (11)	0.0272 (4)	
N1B	0.55655 (18)	0.96259 (14)	0.59078 (12)	0.0204 (4)	
N2B	0.67979 (18)	0.96233 (14)	0.53497 (12)	0.0199 (4)	
N3B	0.8661 (2)	0.98759 (16)	0.44571 (13)	0.0234 (5)	
N4B	0.83900 (19)	0.88679 (15)	0.46948 (12)	0.0227 (5)	
C1B	0.3228 (2)	0.94956 (18)	0.71665 (14)	0.0203 (5)	
H1BA	0.3510	0.8909	0.7056	0.024*	
C2B	0.2040 (2)	0.95066 (18)	0.77424 (14)	0.0208 (5)	
C3B	0.1595 (2)	1.04026 (18)	0.78779 (14)	0.0213 (5)	
C4B	0.2352 (2)	1.12697 (18)	0.74649 (14)	0.0216 (5)	
H4BA	0.2044	1.1865	0.7554	0.026*	
C5B	0.3578 (2)	1.12518 (18)	0.69152 (14)	0.0210 (5)	
H5BA	0.4115	1.1828	0.6656	0.025*	
C6B	0.4004 (2)	1.03784 (17)	0.67511 (14)	0.0194 (5)	
C7B	0.5282 (2)	1.03830 (18)	0.61510 (14)	0.0198 (5)	
H7BA	0.5877	1.0928	0.5951	0.024*	
C8B	0.7711 (2)	1.03861 (18)	0.48228 (14)	0.0221 (5)	
C9B	0.7242 (2)	0.87318 (18)	0.52375 (14)	0.0208 (5)	
C10B	0.6490 (2)	0.77595 (17)	0.57006 (15)	0.0217 (5)	
H10B	0.5516	0.7886	0.5643	0.026*	
C11B	0.6591 (2)	0.73653 (17)	0.66668 (15)	0.0226 (5)	
C12B	0.7824 (3)	0.73933 (19)	0.69533 (16)	0.0299 (6)	
H12B	0.8604	0.7660	0.6550	0.036*	
C13B	0.7903 (3)	0.7030 (2)	0.78281 (17)	0.0342 (6)	
H13B	0.8738	0.7061	0.8003	0.041*	
C14B	0.6771 (3)	0.66181 (19)	0.84572 (16)	0.0300 (6)	
C15B	0.5549 (3)	0.6571 (2)	0.81633 (17)	0.0348 (7)	
H15B	0.4778	0.6284	0.8566	0.042*	
C16B	0.5456 (3)	0.6942 (2)	0.72841 (16)	0.0318 (6)	
H16B	0.4625	0.6907	0.7107	0.038*	
C17B	0.6842 (3)	0.6274 (2)	0.94214 (16)	0.0355 (7)	
H17C	0.5972	0.5962	0.9741	0.043*	0.785 (6)
H17D	0.6932	0.6867	0.9567	0.043*	0.785 (6)
H17E	0.5945	0.6355	0.9725	0.043*	0.215 (6)
H17F	0.7458	0.6709	0.9510	0.043*	0.215 (6)
C18B	0.7994 (5)	0.5541 (3)	0.9742 (2)	0.0301 (10)	0.785 (6)
H18B	0.8865	0.5840	0.9390	0.036*	0.785 (6)
C19B	0.7853 (5)	0.4535 (3)	0.9638 (3)	0.0417 (12)	0.785 (6)
H19D	0.7824	0.4643	0.9039	0.062*	0.785 (6)
H19E	0.7020	0.4214	0.9992	0.062*	0.785 (6)
H19F	0.8628	0.4111	0.9817	0.062*	0.785 (6)
C20B	0.8060 (6)	0.5394 (4)	1.0684 (2)	0.0390 (11)	0.785 (6)
H20D	0.8794	0.4932	1.0877	0.059*	0.785 (6)
H20E	0.7202	0.5126	1.1042	0.059*	0.785 (6)
H20F	0.8225	0.6030	1.0728	0.059*	0.785 (6)
C18C	0.7281 (19)	0.5229 (13)	0.9807 (9)	0.032 (4)	0.215 (6)
H18C	0.6539	0.4816	0.9789	0.038*	0.215 (6)
C19C	0.855 (2)	0.4920 (12)	0.9330 (10)	0.0417 (12)	0.215 (6)
H19G	0.8422	0.5027	0.8746	0.062*	0.215 (6)
H19H	0.8741	0.4219	0.9621	0.062*	0.215 (6)
H19I	0.9313	0.5315	0.9314	0.062*	0.215 (6)
C20C	0.7302 (17)	0.5010 (13)	1.0787 (9)	0.037 (4)	0.215 (6)
H20G	0.7433	0.4300	1.1077	0.056*	0.215 (6)
H20H	0.6443	0.5222	1.1045	0.056*	0.215 (6)
H20I	0.8043	0.5373	1.0845	0.056*	0.215 (6)
C21B	0.1587 (3)	0.77692 (19)	0.80815 (18)	0.0338 (6)	
H21D	0.0948	0.7253	0.8454	0.051*	
H21E	0.2500	0.7570	0.8206	0.051*	
H21F	0.1560	0.7865	0.7485	0.051*	
C22B	0.7028 (3)	0.69751 (19)	0.52920 (17)	0.0308 (6)	
H22D	0.6911	0.7221	0.4692	0.046*	
H22E	0.6525	0.6361	0.5596	0.046*	
H22F	0.7988	0.6849	0.5331	0.046*	
H1N3	0.001 (3)	0.837 (2)	0.3856 (18)	0.040 (9)*	
H2N3	0.938 (3)	1.0140 (19)	0.4079 (16)	0.029 (7)*	
H1O2	0.912 (3)	1.111 (2)	−0.0781 (19)	0.036 (9)*	
H2O2	0.006 (3)	0.984 (2)	0.8625 (19)	0.047 (10)*	

### Atomic displacement parameters (Å^2^)

**Table:**

	*U*^11^	*U*^22^	*U*^33^	*U*^12^	*U*^13^	*U*^23^
S1A	0.0174 (3)	0.0280 (4)	0.0254 (3)	0.0006 (3)	0.0021 (3)	−0.0098 (3)
O1A	0.0181 (9)	0.0224 (9)	0.0300 (9)	0.0023 (7)	0.0054 (7)	−0.0103 (8)
O2A	0.0225 (10)	0.0256 (10)	0.0305 (10)	−0.0032 (8)	0.0067 (8)	−0.0081 (9)
N1A	0.0126 (10)	0.0290 (12)	0.0183 (10)	−0.0001 (9)	−0.0002 (8)	−0.0056 (9)
N2A	0.0122 (10)	0.0237 (11)	0.0185 (10)	0.0005 (8)	−0.0001 (8)	−0.0052 (9)
N3A	0.0166 (11)	0.0283 (12)	0.0202 (11)	−0.0001 (9)	0.0020 (9)	−0.0082 (10)
N4A	0.0177 (11)	0.0279 (12)	0.0252 (11)	−0.0002 (9)	−0.0014 (9)	−0.0086 (9)
C1A	0.0180 (13)	0.0200 (13)	0.0196 (12)	−0.0012 (10)	−0.0044 (10)	−0.0042 (10)
C2A	0.0155 (12)	0.0244 (14)	0.0182 (12)	0.0041 (10)	−0.0033 (10)	−0.0070 (10)
C3A	0.0165 (12)	0.0244 (14)	0.0154 (12)	−0.0013 (10)	−0.0016 (10)	−0.0032 (10)
C4A	0.0242 (13)	0.0199 (13)	0.0219 (13)	0.0006 (10)	−0.0031 (10)	−0.0051 (11)
C5A	0.0200 (13)	0.0282 (14)	0.0234 (13)	0.0054 (11)	−0.0031 (10)	−0.0121 (11)
C6A	0.0164 (12)	0.0251 (14)	0.0168 (12)	0.0011 (10)	−0.0029 (10)	−0.0068 (10)
C7A	0.0181 (13)	0.0256 (15)	0.0206 (13)	0.0030 (11)	−0.0025 (10)	−0.0076 (11)
C8A	0.0124 (12)	0.0291 (14)	0.0169 (12)	0.0024 (10)	−0.0031 (9)	−0.0057 (11)
C9A	0.0162 (12)	0.0293 (15)	0.0189 (12)	0.0011 (10)	−0.0044 (10)	−0.0061 (11)
C10A	0.0226 (13)	0.0274 (14)	0.0229 (13)	0.0004 (11)	−0.0007 (10)	−0.0097 (11)
C11A	0.0205 (13)	0.0176 (13)	0.0255 (13)	0.0002 (10)	−0.0026 (10)	−0.0077 (11)
C12A	0.0214 (13)	0.0222 (13)	0.0251 (13)	−0.0028 (10)	0.0016 (10)	−0.0070 (11)
C13A	0.0276 (14)	0.0207 (13)	0.0242 (13)	−0.0006 (11)	−0.0056 (11)	−0.0044 (11)
C14A	0.0237 (13)	0.0161 (13)	0.0332 (15)	−0.0005 (10)	−0.0043 (11)	−0.0091 (11)
C15A	0.0197 (13)	0.0234 (14)	0.0355 (15)	0.0002 (11)	0.0022 (11)	−0.0113 (12)
C16A	0.0251 (14)	0.0216 (13)	0.0235 (13)	0.0016 (11)	−0.0013 (11)	−0.0054 (11)
C17A	0.0227 (14)	0.0287 (15)	0.0391 (16)	0.0019 (11)	−0.0075 (11)	−0.0126 (12)
C18A	0.0221 (13)	0.0214 (13)	0.0270 (14)	0.0002 (10)	−0.0030 (11)	−0.0047 (11)
C19A	0.0286 (15)	0.0276 (15)	0.0407 (16)	0.0033 (12)	−0.0094 (12)	−0.0062 (13)
C20A	0.0474 (18)	0.0223 (14)	0.0339 (16)	0.0079 (13)	−0.0063 (13)	−0.0067 (12)
C21A	0.0244 (14)	0.0223 (14)	0.0457 (17)	0.0024 (11)	0.0032 (12)	−0.0146 (13)
C22A	0.0279 (15)	0.0364 (16)	0.0415 (16)	0.0011 (12)	−0.0054 (12)	−0.0205 (13)
S1B	0.0273 (4)	0.0275 (4)	0.0281 (4)	−0.0055 (3)	0.0048 (3)	−0.0069 (3)
O1B	0.0181 (9)	0.0238 (9)	0.0265 (9)	−0.0047 (7)	0.0018 (7)	−0.0029 (8)
O2B	0.0165 (9)	0.0348 (12)	0.0268 (10)	−0.0016 (8)	0.0063 (7)	−0.0111 (9)
N1B	0.0113 (10)	0.0276 (12)	0.0195 (10)	0.0002 (8)	0.0012 (8)	−0.0066 (9)
N2B	0.0137 (10)	0.0256 (11)	0.0180 (10)	−0.0003 (8)	0.0002 (8)	−0.0060 (9)
N3B	0.0136 (11)	0.0312 (13)	0.0231 (11)	−0.0010 (9)	0.0028 (9)	−0.0091 (10)
N4B	0.0170 (11)	0.0295 (12)	0.0210 (11)	0.0023 (9)	−0.0023 (8)	−0.0090 (9)
C1B	0.0176 (12)	0.0220 (13)	0.0207 (12)	0.0026 (10)	−0.0024 (10)	−0.0075 (11)
C2B	0.0157 (12)	0.0247 (14)	0.0189 (12)	−0.0031 (10)	−0.0044 (10)	−0.0028 (11)
C3B	0.0165 (12)	0.0291 (14)	0.0157 (12)	0.0016 (10)	−0.0015 (10)	−0.0058 (11)
C4B	0.0213 (13)	0.0238 (14)	0.0205 (12)	0.0031 (10)	−0.0029 (10)	−0.0098 (11)
C5B	0.0192 (13)	0.0226 (13)	0.0189 (12)	−0.0015 (10)	−0.0031 (10)	−0.0041 (10)
C6B	0.0157 (12)	0.0240 (13)	0.0159 (12)	−0.0002 (10)	−0.0033 (9)	−0.0036 (10)
C7B	0.0159 (12)	0.0237 (13)	0.0181 (12)	0.0003 (10)	−0.0030 (10)	−0.0054 (11)
C8B	0.0147 (12)	0.0332 (15)	0.0166 (12)	−0.0026 (11)	−0.0011 (10)	−0.0068 (11)
C9B	0.0164 (12)	0.0277 (14)	0.0202 (12)	0.0062 (10)	−0.0058 (10)	−0.0106 (11)
C10B	0.0165 (12)	0.0245 (14)	0.0241 (13)	0.0011 (10)	−0.0037 (10)	−0.0088 (11)
C11B	0.0218 (13)	0.0182 (13)	0.0267 (13)	0.0046 (10)	−0.0034 (10)	−0.0078 (11)
C12B	0.0258 (14)	0.0330 (15)	0.0261 (14)	−0.0036 (12)	−0.0055 (11)	−0.0034 (12)
C13B	0.0354 (16)	0.0362 (16)	0.0307 (15)	−0.0044 (13)	−0.0133 (12)	−0.0070 (13)
C14B	0.0360 (16)	0.0268 (15)	0.0279 (14)	0.0054 (12)	−0.0042 (12)	−0.0120 (12)
C15B	0.0288 (15)	0.0400 (17)	0.0271 (15)	0.0015 (12)	0.0040 (12)	−0.0059 (13)
C16B	0.0204 (14)	0.0397 (16)	0.0315 (15)	0.0012 (12)	−0.0015 (11)	−0.0095 (13)
C17B	0.0476 (18)	0.0315 (16)	0.0265 (14)	0.0080 (13)	−0.0068 (13)	−0.0103 (12)
C18B	0.038 (3)	0.026 (2)	0.025 (2)	−0.004 (2)	−0.0014 (19)	−0.0080 (17)
C19B	0.069 (3)	0.025 (2)	0.031 (2)	0.004 (2)	−0.007 (2)	−0.0104 (19)
C20B	0.053 (3)	0.036 (3)	0.026 (2)	0.000 (2)	−0.009 (2)	−0.0090 (19)
C18C	0.030 (9)	0.030 (10)	0.030 (8)	0.007 (7)	−0.013 (7)	−0.001 (7)
C19C	0.069 (3)	0.025 (2)	0.031 (2)	0.004 (2)	−0.007 (2)	−0.0104 (19)
C20C	0.030 (9)	0.028 (9)	0.036 (8)	0.013 (7)	−0.008 (7)	0.008 (7)
C21B	0.0279 (15)	0.0279 (15)	0.0401 (16)	−0.0082 (12)	0.0020 (12)	−0.0075 (13)
C22B	0.0331 (15)	0.0316 (15)	0.0297 (15)	0.0060 (12)	−0.0090 (12)	−0.0124 (12)

### Geometric parameters (Å, °)

**Table:**

S1A—C8A	1.680 (2)	N1B—N2B	1.396 (2)
O1A—C2A	1.379 (3)	N2B—C9B	1.376 (3)
O1A—C21A	1.425 (3)	N2B—C8B	1.392 (3)
O2A—C3A	1.366 (3)	N3B—C8B	1.339 (3)
O2A—H1O2	0.75 (3)	N3B—N4B	1.374 (3)
N1A—C7A	1.281 (3)	N3B—H2N3	0.87 (3)
N1A—N2A	1.405 (2)	N4B—C9B	1.298 (3)
N2A—C8A	1.383 (3)	C1B—C2B	1.381 (3)
N2A—C9A	1.383 (3)	C1B—C6B	1.402 (3)
N3A—C8A	1.337 (3)	C1B—H1BA	0.9300
N3A—N4A	1.379 (3)	C2B—C3B	1.398 (3)
N3A—H1N3	0.81 (3)	C3B—C4B	1.375 (3)
N4A—C9A	1.306 (3)	C4B—C5B	1.388 (3)
C1A—C2A	1.375 (3)	C4B—H4BA	0.9300
C1A—C6A	1.403 (3)	C5B—C6B	1.385 (3)
C1A—H1AA	0.9300	C5B—H5BA	0.9300
C2A—C3A	1.403 (3)	C6B—C7B	1.465 (3)
C3A—C4A	1.383 (3)	C7B—H7BA	0.9300
C4A—C5A	1.387 (3)	C9B—C10B	1.495 (3)
C4A—H4AA	0.9300	C10B—C22B	1.523 (3)
C5A—C6A	1.391 (3)	C10B—C11B	1.524 (3)
C5A—H5AA	0.9300	C10B—H10B	0.9800
C6A—C7A	1.458 (3)	C11B—C16B	1.387 (3)
C7A—H7AA	0.9300	C11B—C12B	1.390 (3)
C9A—C10A	1.488 (3)	C12B—C13B	1.377 (3)
C10A—C11A	1.530 (3)	C12B—H12B	0.9300
C10A—C22A	1.530 (3)	C13B—C14B	1.389 (4)
C10A—H10A	0.9800	C13B—H13B	0.9300
C11A—C12A	1.385 (3)	C14B—C15B	1.393 (4)
C11A—C16A	1.396 (3)	C14B—C17B	1.517 (3)
C12A—C13A	1.383 (3)	C15B—C16B	1.387 (4)
C12A—H12A	0.9300	C15B—H15B	0.9300
C13A—C14A	1.383 (3)	C16B—H16B	0.9300
C13A—H13A	0.9300	C17B—C18C	1.460 (15)
C14A—C15A	1.393 (3)	C17B—C18B	1.520 (5)
C14A—C17A	1.516 (3)	C17B—H17C	0.9700
C15A—C16A	1.380 (3)	C17B—H17D	0.9700
C15A—H15A	0.9300	C17B—H17E	0.9602
C16A—H16A	0.9300	C17B—H17F	0.9599
C17A—C18A	1.535 (3)	C18B—C19B	1.518 (6)
C17A—H17A	0.9700	C18B—C20B	1.521 (5)
C17A—H17B	0.9700	C18B—H18B	0.9800
C18A—C20A	1.517 (3)	C19B—H19D	0.9600
C18A—C19A	1.521 (3)	C19B—H19E	0.9600
C18A—H18A	0.9800	C19B—H19F	0.9600
C19A—H19A	0.9600	C20B—H20D	0.9600
C19A—H19B	0.9600	C20B—H20E	0.9600
C19A—H19C	0.9600	C20B—H20F	0.9600
C20A—H20A	0.9600	C18C—C19C	1.49 (2)
C20A—H20B	0.9600	C18C—C20C	1.55 (2)
C20A—H20C	0.9600	C18C—H18C	0.9800
C21A—H21A	0.9600	C19C—H19G	0.9600
C21A—H21B	0.9600	C19C—H19H	0.9600
C21A—H21C	0.9600	C19C—H19I	0.9600
C22A—H22A	0.9600	C20C—H20G	0.9600
C22A—H22B	0.9600	C20C—H20H	0.9600
C22A—H22C	0.9600	C20C—H20I	0.9600
S1B—C8B	1.673 (3)	C21B—H21D	0.9600
O1B—C2B	1.374 (3)	C21B—H21E	0.9600
O1B—C21B	1.428 (3)	C21B—H21F	0.9600
O2B—C3B	1.375 (3)	C22B—H22D	0.9600
O2B—H2O2	0.83 (3)	C22B—H22E	0.9600
N1B—C7B	1.276 (3)	C22B—H22F	0.9600
			
C2A—O1A—C21A	117.01 (17)	C2B—C1B—H1BA	120.4
C3A—O2A—H1O2	104 (2)	C6B—C1B—H1BA	120.4
C7A—N1A—N2A	118.84 (19)	O1B—C2B—C1B	125.8 (2)
C8A—N2A—C9A	108.79 (18)	O1B—C2B—C3B	114.27 (19)
C8A—N2A—N1A	133.50 (19)	C1B—C2B—C3B	119.9 (2)
C9A—N2A—N1A	117.71 (18)	O2B—C3B—C4B	119.7 (2)
C8A—N3A—N4A	114.89 (19)	O2B—C3B—C2B	119.6 (2)
C8A—N3A—H1N3	126 (2)	C4B—C3B—C2B	120.7 (2)
N4A—N3A—H1N3	118 (2)	C3B—C4B—C5B	119.6 (2)
C9A—N4A—N3A	103.57 (19)	C3B—C4B—H4BA	120.2
C2A—C1A—C6A	120.0 (2)	C5B—C4B—H4BA	120.2
C2A—C1A—H1AA	120.0	C6B—C5B—C4B	120.2 (2)
C6A—C1A—H1AA	120.0	C6B—C5B—H5BA	119.9
C1A—C2A—O1A	125.1 (2)	C4B—C5B—H5BA	119.9
C1A—C2A—C3A	120.3 (2)	C5B—C6B—C1B	120.2 (2)
O1A—C2A—C3A	114.67 (19)	C5B—C6B—C7B	119.3 (2)
O2A—C3A—C4A	117.9 (2)	C1B—C6B—C7B	120.4 (2)
O2A—C3A—C2A	122.3 (2)	N1B—C7B—C6B	118.8 (2)
C4A—C3A—C2A	119.8 (2)	N1B—C7B—H7BA	120.6
C3A—C4A—C5A	119.9 (2)	C6B—C7B—H7BA	120.6
C3A—C4A—H4AA	120.0	N3B—C8B—N2B	101.5 (2)
C5A—C4A—H4AA	120.0	N3B—C8B—S1B	126.27 (18)
C4A—C5A—C6A	120.5 (2)	N2B—C8B—S1B	132.20 (17)
C4A—C5A—H5AA	119.7	N4B—C9B—N2B	110.2 (2)
C6A—C5A—H5AA	119.7	N4B—C9B—C10B	126.5 (2)
C5A—C6A—C1A	119.4 (2)	N2B—C9B—C10B	123.29 (19)
C5A—C6A—C7A	118.6 (2)	C9B—C10B—C22B	110.97 (19)
C1A—C6A—C7A	121.9 (2)	C9B—C10B—C11B	110.98 (19)
N1A—C7A—C6A	120.1 (2)	C22B—C10B—C11B	110.61 (19)
N1A—C7A—H7AA	120.0	C9B—C10B—H10B	108.1
C6A—C7A—H7AA	120.0	C22B—C10B—H10B	108.1
N3A—C8A—N2A	102.15 (19)	C11B—C10B—H10B	108.1
N3A—C8A—S1A	127.63 (17)	C16B—C11B—C12B	118.2 (2)
N2A—C8A—S1A	130.20 (18)	C16B—C11B—C10B	120.5 (2)
N4A—C9A—N2A	110.6 (2)	C12B—C11B—C10B	121.3 (2)
N4A—C9A—C10A	125.6 (2)	C13B—C12B—C11B	120.7 (2)
N2A—C9A—C10A	123.8 (2)	C13B—C12B—H12B	119.6
C9A—C10A—C11A	110.58 (19)	C11B—C12B—H12B	119.6
C9A—C10A—C22A	110.9 (2)	C12B—C13B—C14B	122.0 (2)
C11A—C10A—C22A	110.7 (2)	C12B—C13B—H13B	119.0
C9A—C10A—H10A	108.2	C14B—C13B—H13B	119.0
C11A—C10A—H10A	108.2	C13B—C14B—C15B	116.9 (2)
C22A—C10A—H10A	108.2	C13B—C14B—C17B	121.7 (2)
C12A—C11A—C16A	118.0 (2)	C15B—C14B—C17B	121.3 (2)
C12A—C11A—C10A	121.1 (2)	C16B—C15B—C14B	121.5 (2)
C16A—C11A—C10A	120.8 (2)	C16B—C15B—H15B	119.2
C13A—C12A—C11A	120.7 (2)	C14B—C15B—H15B	119.2
C13A—C12A—H12A	119.6	C11B—C16B—C15B	120.7 (2)
C11A—C12A—H12A	119.6	C11B—C16B—H16B	119.7
C14A—C13A—C12A	121.8 (2)	C15B—C16B—H16B	119.7
C14A—C13A—H13A	119.1	C18C—C17B—C14B	114.9 (6)
C12A—C13A—H13A	119.1	C18C—C17B—C18B	32.2 (7)
C13A—C14A—C15A	117.3 (2)	C14B—C17B—C18B	116.8 (2)
C13A—C14A—C17A	120.7 (2)	C18C—C17B—H17C	79.2
C15A—C14A—C17A	122.0 (2)	C14B—C17B—H17C	108.1
C16A—C15A—C14A	121.5 (2)	C18B—C17B—H17C	108.1
C16A—C15A—H15A	119.2	C18C—C17B—H17D	131.9
C14A—C15A—H15A	119.2	C14B—C17B—H17D	108.1
C15A—C16A—C11A	120.6 (2)	C18B—C17B—H17D	108.1
C15A—C16A—H16A	119.7	H17C—C17B—H17D	107.3
C11A—C16A—H16A	119.7	C18C—C17B—H17E	108.1
C14A—C17A—C18A	113.7 (2)	C14B—C17B—H17E	108.5
C14A—C17A—H17A	108.8	C18B—C17B—H17E	129.5
C18A—C17A—H17A	108.8	H17C—C17B—H17E	33.0
C14A—C17A—H17B	108.8	H17D—C17B—H17E	76.2
C18A—C17A—H17B	108.8	C18C—C17B—H17F	109.2
H17A—C17A—H17B	107.7	C14B—C17B—H17F	108.5
C20A—C18A—C19A	110.5 (2)	C18B—C17B—H17F	78.9
C20A—C18A—C17A	112.2 (2)	H17C—C17B—H17F	133.8
C19A—C18A—C17A	110.2 (2)	H17D—C17B—H17F	33.6
C20A—C18A—H18A	108.0	H17E—C17B—H17F	107.5
C19A—C18A—H18A	108.0	C19B—C18B—C17B	112.3 (4)
C17A—C18A—H18A	108.0	C19B—C18B—C20B	110.8 (3)
C18A—C19A—H19A	109.5	C17B—C18B—C20B	110.4 (3)
C18A—C19A—H19B	109.5	C19B—C18B—H18B	107.7
H19A—C19A—H19B	109.5	C17B—C18B—H18B	107.7
C18A—C19A—H19C	109.5	C20B—C18B—H18B	107.7
H19A—C19A—H19C	109.5	C17B—C18C—C19C	115.6 (14)
H19B—C19A—H19C	109.5	C17B—C18C—C20C	107.9 (11)
C18A—C20A—H20A	109.5	C19C—C18C—C20C	115.0 (12)
C18A—C20A—H20B	109.5	C17B—C18C—H18C	105.8
H20A—C20A—H20B	109.5	C19C—C18C—H18C	105.8
C18A—C20A—H20C	109.5	C20C—C18C—H18C	105.8
H20A—C20A—H20C	109.5	C18C—C19C—H19G	109.5
H20B—C20A—H20C	109.5	C18C—C19C—H19H	109.5
O1A—C21A—H21A	109.5	H19G—C19C—H19H	109.5
O1A—C21A—H21B	109.5	C18C—C19C—H19I	109.5
H21A—C21A—H21B	109.5	H19G—C19C—H19I	109.5
O1A—C21A—H21C	109.5	H19H—C19C—H19I	109.5
H21A—C21A—H21C	109.5	C18C—C20C—H20G	109.5
H21B—C21A—H21C	109.5	C18C—C20C—H20H	109.5
C10A—C22A—H22A	109.5	H20G—C20C—H20H	109.5
C10A—C22A—H22B	109.5	C18C—C20C—H20I	109.5
H22A—C22A—H22B	109.5	H20G—C20C—H20I	109.5
C10A—C22A—H22C	109.5	H20H—C20C—H20I	109.5
H22A—C22A—H22C	109.5	O1B—C21B—H21D	109.5
H22B—C22A—H22C	109.5	O1B—C21B—H21E	109.5
C2B—O1B—C21B	116.45 (18)	H21D—C21B—H21E	109.5
C3B—O2B—H2O2	108 (2)	O1B—C21B—H21F	109.5
C7B—N1B—N2B	118.68 (19)	H21D—C21B—H21F	109.5
C9B—N2B—C8B	109.26 (18)	H21E—C21B—H21F	109.5
C9B—N2B—N1B	118.38 (18)	C10B—C22B—H22D	109.5
C8B—N2B—N1B	132.31 (19)	C10B—C22B—H22E	109.5
C8B—N3B—N4B	114.66 (19)	H22D—C22B—H22E	109.5
C8B—N3B—H2N3	125.0 (17)	C10B—C22B—H22F	109.5
N4B—N3B—H2N3	120.3 (17)	H22D—C22B—H22F	109.5
C9B—N4B—N3B	104.41 (18)	H22E—C22B—H22F	109.5
C2B—C1B—C6B	119.3 (2)		
			
C7A—N1A—N2A—C8A	−0.3 (3)	C21B—O1B—C2B—C3B	176.7 (2)
C7A—N1A—N2A—C9A	179.9 (2)	C6B—C1B—C2B—O1B	−176.3 (2)
C8A—N3A—N4A—C9A	0.5 (3)	C6B—C1B—C2B—C3B	2.6 (3)
C6A—C1A—C2A—O1A	179.2 (2)	O1B—C2B—C3B—O2B	−2.5 (3)
C6A—C1A—C2A—C3A	−1.0 (3)	C1B—C2B—C3B—O2B	178.5 (2)
C21A—O1A—C2A—C1A	−6.5 (3)	O1B—C2B—C3B—C4B	176.8 (2)
C21A—O1A—C2A—C3A	173.7 (2)	C1B—C2B—C3B—C4B	−2.2 (3)
C1A—C2A—C3A—O2A	179.4 (2)	O2B—C3B—C4B—C5B	178.6 (2)
O1A—C2A—C3A—O2A	−0.7 (3)	C2B—C3B—C4B—C5B	−0.7 (3)
C1A—C2A—C3A—C4A	0.4 (3)	C3B—C4B—C5B—C6B	3.1 (3)
O1A—C2A—C3A—C4A	−179.8 (2)	C4B—C5B—C6B—C1B	−2.6 (3)
O2A—C3A—C4A—C5A	−178.4 (2)	C4B—C5B—C6B—C7B	178.4 (2)
C2A—C3A—C4A—C5A	0.8 (3)	C2B—C1B—C6B—C5B	−0.3 (3)
C3A—C4A—C5A—C6A	−1.2 (3)	C2B—C1B—C6B—C7B	178.7 (2)
C4A—C5A—C6A—C1A	0.6 (3)	N2B—N1B—C7B—C6B	−178.74 (19)
C4A—C5A—C6A—C7A	179.5 (2)	C5B—C6B—C7B—N1B	−170.1 (2)
C2A—C1A—C6A—C5A	0.5 (3)	C1B—C6B—C7B—N1B	10.9 (3)
C2A—C1A—C6A—C7A	−178.3 (2)	N4B—N3B—C8B—N2B	1.8 (2)
N2A—N1A—C7A—C6A	178.74 (19)	N4B—N3B—C8B—S1B	−175.74 (17)
C5A—C6A—C7A—N1A	175.1 (2)	C9B—N2B—C8B—N3B	−2.0 (2)
C1A—C6A—C7A—N1A	−6.1 (3)	N1B—N2B—C8B—N3B	−179.3 (2)
N4A—N3A—C8A—N2A	−0.7 (2)	C9B—N2B—C8B—S1B	175.30 (19)
N4A—N3A—C8A—S1A	177.76 (17)	N1B—N2B—C8B—S1B	−2.0 (4)
C9A—N2A—C8A—N3A	0.6 (2)	N3B—N4B—C9B—N2B	−0.5 (2)
N1A—N2A—C8A—N3A	−179.3 (2)	N3B—N4B—C9B—C10B	−179.5 (2)
C9A—N2A—C8A—S1A	−177.78 (18)	C8B—N2B—C9B—N4B	1.7 (3)
N1A—N2A—C8A—S1A	2.3 (4)	N1B—N2B—C9B—N4B	179.41 (18)
N3A—N4A—C9A—N2A	−0.1 (2)	C8B—N2B—C9B—C10B	−179.4 (2)
N3A—N4A—C9A—C10A	176.8 (2)	N1B—N2B—C9B—C10B	−1.6 (3)
C8A—N2A—C9A—N4A	−0.4 (3)	N4B—C9B—C10B—C22B	−14.6 (3)
N1A—N2A—C9A—N4A	179.54 (18)	N2B—C9B—C10B—C22B	166.5 (2)
C8A—N2A—C9A—C10A	−177.3 (2)	N4B—C9B—C10B—C11B	108.8 (3)
N1A—N2A—C9A—C10A	2.6 (3)	N2B—C9B—C10B—C11B	−70.0 (3)
N4A—C9A—C10A—C11A	−105.8 (3)	C9B—C10B—C11B—C16B	139.1 (2)
N2A—C9A—C10A—C11A	70.7 (3)	C22B—C10B—C11B—C16B	−97.3 (3)
N4A—C9A—C10A—C22A	17.4 (3)	C9B—C10B—C11B—C12B	−42.7 (3)
N2A—C9A—C10A—C22A	−166.1 (2)	C22B—C10B—C11B—C12B	80.9 (3)
C9A—C10A—C11A—C12A	50.2 (3)	C16B—C11B—C12B—C13B	−1.4 (4)
C22A—C10A—C11A—C12A	−73.0 (3)	C10B—C11B—C12B—C13B	−179.7 (2)
C9A—C10A—C11A—C16A	−132.4 (2)	C11B—C12B—C13B—C14B	0.4 (4)
C22A—C10A—C11A—C16A	104.3 (3)	C12B—C13B—C14B—C15B	1.1 (4)
C16A—C11A—C12A—C13A	−0.7 (3)	C12B—C13B—C14B—C17B	−176.4 (2)
C10A—C11A—C12A—C13A	176.7 (2)	C13B—C14B—C15B—C16B	−1.6 (4)
C11A—C12A—C13A—C14A	−0.5 (4)	C17B—C14B—C15B—C16B	175.9 (2)
C12A—C13A—C14A—C15A	1.5 (3)	C12B—C11B—C16B—C15B	0.9 (4)
C12A—C13A—C14A—C17A	−176.4 (2)	C10B—C11B—C16B—C15B	179.2 (2)
C13A—C14A—C15A—C16A	−1.2 (3)	C14B—C15B—C16B—C11B	0.6 (4)
C17A—C14A—C15A—C16A	176.6 (2)	C13B—C14B—C17B—C18C	−89.0 (9)
C14A—C15A—C16A—C11A	0.0 (4)	C15B—C14B—C17B—C18C	93.6 (9)
C12A—C11A—C16A—C15A	1.0 (3)	C13B—C14B—C17B—C18B	−53.2 (4)
C10A—C11A—C16A—C15A	−176.5 (2)	C15B—C14B—C17B—C18B	129.4 (3)
C13A—C14A—C17A—C18A	89.9 (3)	C18C—C17B—C18B—C19B	30.3 (11)
C15A—C14A—C17A—C18A	−87.9 (3)	C14B—C17B—C18B—C19B	−64.4 (5)
C14A—C17A—C18A—C20A	66.0 (3)	C18C—C17B—C18B—C20B	−93.9 (12)
C14A—C17A—C18A—C19A	−170.5 (2)	C14B—C17B—C18B—C20B	171.4 (3)
C7B—N1B—N2B—C9B	166.3 (2)	C14B—C17B—C18C—C19C	49.4 (18)
C7B—N1B—N2B—C8B	−16.6 (3)	C18B—C17B—C18C—C19C	−51.9 (14)
C8B—N3B—N4B—C9B	−0.9 (3)	C14B—C17B—C18C—C20C	179.7 (9)
C21B—O1B—C2B—C1B	−4.4 (3)	C18B—C17B—C18C—C20C	78.4 (14)

### Hydrogen-bond geometry (Å, °)

**Table:**

Cg1 is the centroid of the C11A—C16A phenyl ring.

**Table:**

*D*—H···*A*	*D*—H	H···*A*	*D*···*A*	*D*—H···*A*
N3A—H1N3···N4B^i^	0.82 (3)	2.19 (3)	2.968 (3)	161 (3)
N3B—H2N3···S1A^ii^	0.87 (3)	2.39 (3)	3.227 (2)	161 (3)
O2A—H1O2···O2B^iii^	0.75 (3)	2.11 (3)	2.808 (3)	157 (3)
C7A—H7AA···S1A	0.93	2.43	3.193 (2)	139
C7B—H7BA···S1B	0.93	2.53	3.243 (2)	134
C5B—H5BA···Cg1^iv^	0.93	2.91	3.660 (3)	139

Symmetry codes: (i) *x*−1, *y*, *z*; (ii) *x*+1, *y*, *z*; (iii) *x*+1, *y*, *z*−1; (iv) −*x*+1, −*y*+2, −*z*+1.
